# Early safety and effectiveness of new 3‑dimensional lightweight SWING‑Mesh in minimally‑invasive inguinal hernia repair: a multicenter prospective observational study with 3‑month follow‑up

**DOI:** 10.20452/wiitm.2025.18012

**Published:** 2026-02-03

**Authors:** Mateusz Zamkowski, Maciej Bękarski, Jacek Białecki, Jarosław Chudek, Grzegorz Dobkowski, Paula Franczak, Jacek Grabias, Jakub Gołyski, Krystian Kisielewski, Marcin Kontek, Aleksander Król, Volodymyr Lavrynets, Bartosz Molasy, Kryspin Mitura, Dorota Piątek‑Czypionka, Michał Putko, Jerzy Ropel, Przemysław Rymkiewicz, Sławomir Saluk, Leszek Sułkowski, Dominik Wieczorek, Marcin Włodarczyk, Marta Wójcik, Maciej Śmietański

**Affiliations:** Department of Śmietański Hernia Center, Luxmed Hospital in Gdansk, Gdańsk, Poland; Department of Second Department of Radiology Medical University of Gdansk Gdansk Poland; Department of General, Vascular, and Oncological Surgery, Mazovian Brodnowski Hospital, Warszawa, Poland; Department of General and Vascular Surgery, Faculty of Medicine Medical University of Warsaw Warszawa Poland; Department of General and Minimally-Invasive Surgery, Franciszek Raszeja Municipal Hospital in Poznan, Poznań, Poland; Department of General and Oncological Surgery, Independent Public Health Care Centre in Brzesko, Brzesko, Poland; Department of General Surgery Military Institute of Aviation Medicine Warszawa Poland; Department of General and Oncological Surgery, Florian Ceynowa Specialist Hospital, Gdańsk, Poland; Department of General and Oncological Surgery, Prof. S. T. Dąbrowski Hospital, Puszczykowo, Poland; Department of Oncological and Minimally-Invasive Surgery, St. John of Dukla Oncology Center of the Lublin Region, Lublin, Poland; Department of General Surgery, Municipal Hospital SPZOZ in Siedlce, Siedlce, Poland; Department of General Surgery, Dr. A. Sokołowski Specialist Hospital in Wałbrzych, Wałbrzych, Poland; Department of General Surgery, Karol Jonscher Municipal Medical Centre in Lodz, Lodz, Poland; Department of General Surgery, Nowy Szpital in Kostrzyn nad Odrą, Kostrzyn nad Odrą, Poland; Department of General and Colorectal Surgery, St. Alexander Hospital in Kielce, Kielce, Poland; Department of Collegium Medicum, Jan Kochanowski University in Kielce, Kielce, Poland; Department of General Surgery, Provincial Specialist Hospital No. 4 in Bytom, Bytom, Poland; Department of General Surgery, Regional Health Center, Kartuzy, Poland; Department of General, Minimally-Invasive, and Elderly Surgery, University of Warmia and Mazury, Olsztyn, Poland; Department of General Surgery, Regional Specialist Hospital, Częstochowa, Poland; Department of General Surgery, District Hospital, Szczytno, Poland; Department of General and Oncological Surgery, Faculty of Medicine Medical University of Lodz Łódź Poland

**Keywords:** 3D mesh, inguinal hernia, laparoendoscopic repair, totally extraperitoneal, transabdominal preperitoneal

## Abstract

**INTRODUCTION:**

Independent postmarket clinical evidence for newly introduced inguinal meshes remains limited. We conducted a multicenter prospective observational study to assess early safety and effectiveness of fixation-free SWING-Mesh in transabdominal preperitoneal (TAPP) / totally extraperitoneal (TEP) repair.

**AIM:**

We aimed to assess early (3-month) safety and effectiveness of fixation-free SWING-Mesh use in TAPP/TEP repair.

**MATERIALS AND METHODS:**

A prospective cohort study was conducted in 20 Polish centers, of which 1 was excluded after central monitoring. Consecutive adults underwent elective TAPP or TEP repair with the unfixed polypropylene SWING-Mesh. Exclusion criteria comprised emergency surgery, bowel resection, contraindications to laparoendoscopic repair, and large direct M3 hernias. Recurrence, complications, unplanned visits / interventions and pain (as per the Visual Analog Scale [VAS]) at discharge, 7–10 days, 30 days, and 3 months postoperatively were recorded. The unit of analysis was the operated groin (case).

**RESULTS:**

We analyzed 294 cases in a total of 283 patients at a mean (SD) age of 51.9 (15.9) years, 84.4% of which were men. TAPP repair was performed in 86.4%, and TEP procedure in 13.6% of the patients. There were no instances of hernia recurrence by 3 months postsurgery. Pain decreased over time (*P* <⁠0.001): mean (SD) VAS score of 1.8 (1.4) at discharge, 1 (1.2) at 7–10 days, 0.5 (1.1) at 30 days, and 0.5 (1) at 3 months postoperatively. At 3 months after the procedure, 78.5% of the individuals reported a VAS score of 0, and 3.1%, a score equal to or greater than 4. Complication rates were below 10% at each time point and were mostly minor; 6.9% or fewer patients required an unplanned visit or intervention by 3 months postoperatively.

**CONCLUSIONS:**

Fixation-free SWING-Mesh use in TAPP/TEP repair was associated with favorable early outcomes. Twelve-month follow-up will report long-term recurrence and chronic pain.

## INTRODUCTION

Over the past decade, the process of introducing new surgical implants into the market has undergone substantial evolution. With the implementation of the European Union Medical Device Regulation, many surgical meshes were reclassified from class II to class III medical devices, reflecting the need for stricter scrutiny and more robust clinical evidence in response to safety concerns arising from specific products, such as Physiomesh (Ethicon, Inc., Raritan, New Jersey, United States), among others.^1^^,^^2^^,^^3^ In everyday practice, however, a surgeon who is to place a new implant is still largely dependent on information provided by distributors and sales representatives, whose obvious commercial interest is to emphasize the advantages of their product. These claims are sometimes supported by animal experiments and only rarely by solid, prospective preclinical, or early clinical data in humans. We are frequently reassured about the reliability of a new material and its exceptional properties, and this narrative is often accepted as fact in the absence of independent verification.

Therefore, under the auspices of the Hernia Chapter of the Association of Polish Surgeons, we initiated a prospective multicenter observational study evaluating fixation-free SWING-Mesh (SMH2, THT Bioscience, Montpellier, France) in routine transabdominal preperitoneal (TAPP) and, totally extraperitoneal (TEP) repair, using consecutive cases and predefined follow-up assessments.

Minimally-invasive inguinal hernia (IH) repair has become a recommended first-line approach in contemporary international guidelines, provided adequate institutional resources and surgical experience are available.^4^^,^^5^ The TAPP and TEP techniques offer shorter convalescence, lower early postoperative pain, reduced wound-related complications, and a potentially lower risk of chronic groin pain than open repair procedures. The transition toward laparoendoscopic approaches has also shifted the focus of mesh design. Flat meshes originally developed for open techniques, such as the Lichtenstein technique, may not optimally conform to the complex 3-dimensional (3D) geometry of the myopectineal orifice (MPO), which resembles a shallow dome with natural curvatures.^6^^,^^7^^,^^8^ In this setting, a planar implant may provide limited contact surface and may be prone to folding or migration under intra-abdominal pressure.

To address these issues, 3D preformed meshes were developed. Their anatomical shape is intended to improve coverage and stability of the MPO, facilitate correct placement, and, in some systems, allow for fixation-free implantation without tacks or staples. Clinical data on 3D meshes in large direct (M3) hernias have suggested good hernia control and favorable pain outcomes even without fixation, encouraging wider adoption of anatomical designs.^8^ SWING-Mesh is a next-generation, lightweight, macroporous 3D polypropylene implant, specifically tailored for laparoendoscopic IH repair. Its 3D form is achieved by cutting and re-fusing polypropylene fibers, eliminating the need for rigid rings or additional support elements, and the symmetrical design allows for its use on either side (right or left groin).

## AIM

To provide independent, real-world evidence on the early safety and effectiveness of the newly introduced SWING-Mesh 3D polypropylene implant used without fixation in laparoendoscopic IH repair, we conducted a prospective multicenter observational study across Polish hernia centers evaluating outcomes after TAPP and TEP repairs. This paper reports early (up to 3-month) results focusing on postoperative pain, complications, and recurrence.

## MATERIALS AND METHODS

### Study design and setting

We conducted a prospective multicenter observational cohort study in 20 surgical centers in Poland; however, 1 center was excluded following data quality monitoring (due to discrepancies), and the final analysis included 19 centers. Consecutive adult patients scheduled for elective laparoendoscopic IH repair using SWING-Mesh were enrolled between April and August 2025. The 12-month follow-up is scheduled to be completed in September 2026. The unit of analysis was the hernia repair (“case”), defined as 1 operated groin. Patients with bilateral IHs contributed 2 cases. The study protocol and statistical analysis plan were defined a priori. The coordinating center was the Śmietański Hernia Center in Gdansk. The study was registered at ClinicalTrials.gov (NCT06915155). Reporting followed the Strengthening the Reporting of Observational Studies in Epidemiology recommendations for observational cohort studies^9^.

**FIGURE 1:**
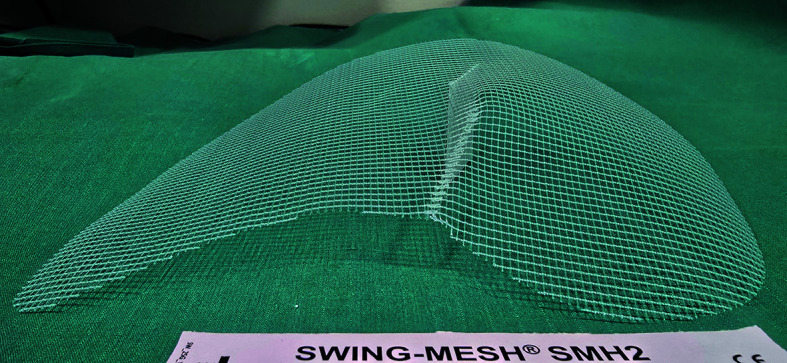
SWING-Mesh: lightweight bidirectional polypropylene mesh with multidirectional elasticity and semirigid curvature, designed for left or right use

**FIGURE 2:**
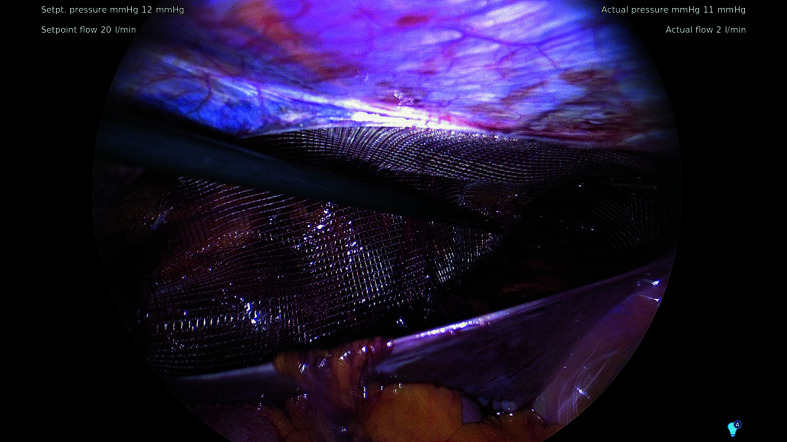
Intraoperative view of the SWING-Mesh implant positioned in the myopectineal orifice during laparoendoscopic inguinal hernia repair

### Eligibility criteria

Patients of both sexes presenting with a primary or recurrent IH and referred for elective laparoendoscopic repair (TAPP or TEP) were screened for inclusion. Eligible participants were required to be 18 years or older, able to provide written informed consent, eligible for general anesthesia and laparoendoscopic surgery, and willing to comply with the scheduled follow-up. Major exclusion criteria (per protocol) were emergency surgery, incarcerated or strangulated hernia requiring bowel resection, and any condition precluding safe laparoendoscopic access. In addition, patients with large direct M3 hernias were excluded from the study, based on previous findings from multicenter MEFISTO (Mesh Fixation Study in Laparoendoscopic Repair of M3 Inguinal Hernias) trial, which indicated that this type of defect may require additional fixation when lightweight meshes are used.^8^^,^^10^ To preserve a uniform, fixation-free concept and avoid systematic underperformance of the implant in this specific subgroup, M3 hernias were therefore not included in the present cohort. This decision is further supported by recent pressure-chamber testing, which suggests that SWING-Mesh may be suboptimal for large direct (M3) defects under high intra-abdominal pressure conditions.^11^

All operations were performed using established TAPP or TEP techniques according to the operating surgeon’s preference and local practice. In each case, a 3D lightweight polypropylene SWING-Mesh implant (15 cm × 11 cm or 16 cm × 12 cm; at the surgeon’s discretion) was placed to cover the MPO and all hernia defects (Figure 1 and Figure 2). No additional mechanical fixation (tacks, staples, sutures, or glue) was used, in line with the design premise that the 3D configuration provides stable self-positioning of the implant. Operative details captured in the study database included the side of repair (right / left / bilateral), operative time, intraoperative complications, use of antibiotic prophylaxis, and the choice of TAPP vs TEP repair. Postoperative drainage was not routinely applied. Given the lack of unequivocal evidence supporting its effectiveness after TEP repair, the decision to place a drain was left to the operating surgeon’s discretion.^12^

### Outcomes

The original study concept defined the primary effectiveness end point as hernia recurrence within a 12-month observation window, with expected recurrence of 2%–4% and a noninferiority margin of 6%. The present analysis focused on 3-month follow-up.

### Safety and patient-reported outcomes

Safety and patient-reported outcomes included: postoperative groin pain intensity measured on a 0–10 Visual Analog Scale (VAS) at discharge, 7–10 days, 30 days, and 3 months postoperatively; early postoperative complications occurring within 30 days (including hematoma, seroma, and wound-related events); later complications recorded up to 3 months postsurgery (including groin discomfort, foreign-body sensation, testicular pain, and seroma); and the need for additional postoperative interventions or unplanned postoperative visits.

In the full study protocol, chronic pain was defined as a VAS score above 4 persisting beyond 3 months and interfering with daily activities, with an expected incidence of less than 5%. Because the present report is limited to the early 3-month follow-up, we present the proportion of patients with a VAS score equal to or greater than 4 at 3 months postoperatively as a surrogate marker of clinically relevant pain. The definitive chronic pain analysis will be reported at 12 months.

**FIGURE 3:**
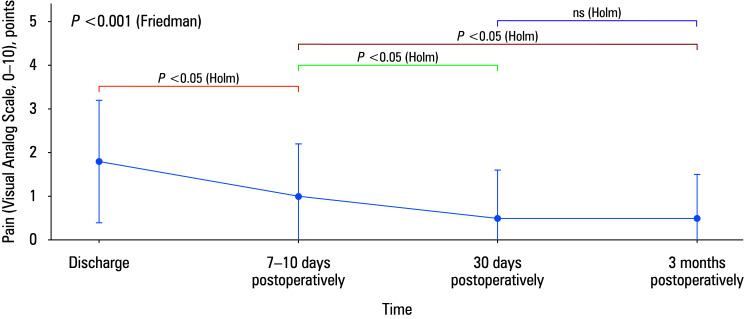
Postoperative groin pain over time assessed by the Visual Analog Scale (0–10) and presented as mean (SD) at discharge, 7–10 days, 30 days, and 3 months postoperatively

Abbreviations: ns, not significant

### Data collection and follow-up

Baseline data included age, sex, body mass index (BMI), comorbidities, occupation, family history of hernia, and smoking status. Peri- and early postoperative data were obtained from hospital records. Follow-up assessments at 7–10 days, 30 days, and 3 months postoperatively were performed in person or via structured telephone interviews, using standardized questionnaires for pain and symptoms, and were carried out by designated study personnel at each participating hospital. All data were centrally monitored by a coordinating committee, which supervised completeness and consistency of the database and double-checked discrepant entries. As a result of this quality control process, 1 center was excluded entirely from the study due to significant data inconsistencies. Clinical examination was used to verify suspected recurrence whenever feasible; in equivocal cases, imaging could be performed at the treating physician’s discretion.

### Sample size

The statistical analysis plan assumed that inclusion of 300 cases would provide a 95% CI for recurrence of approximately 2.3%–6.9%, and 80% power for noninferiority analyses vs historical recurrence rates of 2%–4% with a 6% margin.

The dataset included 294 cases (283 patients)—marginally below this target but still sufficient to provide a precise estimate of early recurrence.

### Statistical analysis

Statistical methods were prespecified in the analysis plan and implemented using Statistica software, version 12.0 (TIBCO Software Inc., Palo Alto, California, United States) and Microsoft Excel (Microsoft, Redmond, Washington, United States). Quantitative variables were summarized primarily as mean (SD) for descriptive reporting, and qualitative variables were presented as counts and percentages. Normality of continuous variables was assessed with the Shapiro–Wilk test, and homogeneity of variance with the Levene / Brown–Forsythe test. Postoperative groin pain (VAS scale of 0–10) was assessed at multiple time points and analyzed as repeated measurements using the Friedman test. Following a significant Friedman test result, post-hoc pairwise comparisons were performed using the Wilcoxon signed-rank test, with adjustment for multiple testing (the Holm method), and the corresponding *P* values are reported in (Figure 3). Changes in categorical outcomes over time (eg, complication rates) were evaluated using χ^2^-based methods, with the Yates correction, the Fisher exact test, or the Cochran criteria, as appropriate. A 2-sided* P* value below 0.05 was considered significant. For recurrence, exact binomial confidence intervals were calculated.

### Ethics

The study was conducted in accordance with the Declaration of Helsinki and applicable local regulations. The protocol was approved by the institutional review board of the coordinating center and, where required, by local ethics committees at participating centers. All patients provided written informed consent prior to inclusion. Ethical approval for the study was granted by the Medical Chamber Ethics Committee in Gdańsk (KB-1022/2025).

## RESULTS

### Study population

During the study period, 294 cases were analyzed, corresponding to 283 patients, of which 11 underwent bilateral repair and therefore contributed 2 cases each. For the purpose of this study, each operated groin was treated as a separate case and analyzed independently. Accordingly, all operative Tables and case-based statistical analyses were constructed at the case level. There was a marked male predominance: 248 patients (84.4%) were men and 46 (15.6%) were women. Mean (SD) age of the cohort was 51.9 (15.9) years. Mean (SD) BMI was 26 (3.6) kg/m^2^. Comorbidities were present in 164 patients (55.8%). Regarding occupation, 45.6% of the participants were office workers and 43.2% manual workers, with smaller proportions of the unemployed and retirees. A positive family history of hernia was reported by 17.8% of the study population, and 15.4% were current smokers (Table 1).

**TABLE 1:** Baseline characteristics of the study population (n = 294)^a^

Characteristic		Value
Sex	Men	248 (84.4)
	Women	46 (15.6)
Age, y		51.9 (15.9)
BMI, kg/m²		26 (3.6)
≥1 comorbidity		164 (55.8)
Occupation	White-collar worker	134 (45.6)
	Manual worker	127 (43.2)
	Unemployed	11 (3.7)
	Other	1 (0.3)
	Retired / on pension	21 (7.1)
Family history of hernia		52 (17.8)
Current smoker		45 (15.4)

Data are presented as mean (SD) or number (percentage).

a 294 cases correspond to 283 patients, as 11 patients underwent bilateral repair.

**TABLE 2:** Operative characteristics

Characteristic			Value
Laterality^a^	Right		149 (50.7)
	Left		134 (45.6)
	Bilateral		11 (3.7)
Operative variables^b^	Operative time, min		66.3 (36.4)
	Intraoperative complications		51 (18)
	Antibiotic prophylaxis		175 (59.5)
	Surgical technique	TAPP	254 (86.4)
		TEP	40 (13.6)

Data are presented as mean (SD) or number (percentage).

**a **Laterality is reported per patient (n = 283). Bilateral repairs contributed 2 cases each.

**b **Operative variables are reported per case (n = 294).

Abbreviations: TAPP, transabdominal preperitoneal; TEP, totally extraperitoneal

**TABLE 3:** Postoperative groin pain assessed by the Visual Analog Scale (0–10 points) at different time points

VAS score, points	At discharge	7–10 days postoperatively	30 days postoperatively	3 months postoperatively
0	50 (17.4)	122 (43)	193 (71)	205 (78.5)
1–3	207 (71.9)	150 (52.8)	72 (26.5)	48 (18.4)
4–7	29 (10.1)	11 (3.9)	7 (2.6)	8 (3.1)
8–10	2 (0.6)	1 (0.4)	0	0
Mean (SD)	1.8 (1.4)	1 (1.2)	0.5 (1.1)	0.5 (1)
Range	0–9	0–8	0–7	0–5

Data are presented as number (percentage) unless indicated otherwise.

Abbreviations: VAS, Visual Analog Scale

### Patient flow and follow-up

A total of 323 cases were initially enrolled in the study. Of these, 29 cases from a single center were excluded due to data inconsistencies identified by the monitoring committee, leaving 294 cases for the present analysis. The number of cases lost to follow-up was 27 (9.2%).

#### Operative details

Right-sided repair was performed in 149 cases (50.7%), left-sided in 134 (45.6%), and bilateral in 11 (3.7%). Mean (SD) operative time was 66.3 (36.4) minutes. Intraoperative complications were recorded in 51 procedures (18%). Antibiotic prophylaxis was administered in 59.5% of the cases. TAPP was the predominant technique, used in 254 operations (86.4%), whereas TEP repair was performed in 40 cases (13.6%; Table 2).

### Pain outcomes

Postoperative pain in the operated groin decreased over time (Friedman test; *P* <⁠0.001). For clinical interpretability, pain intensity was summarized descriptively as mean (SD) at each time point and complemented by categorical VAS distributions. At discharge, mean (SD) VAS score was 1.8 (1.4), with 17.4% of the cases reporting a 0 score, 71.9% reporting a score of 1–3, 10.1% indicating a 4–7 score, and 0.6% reporting a score of 8–10. At 7–10 days postoperatively, mean (SD) VAS score decreased to 1 (1.2), and the distribution shifted toward lower scores (43%, VAS 0; 52.8%, VAS 1–3; 3.9%, VAS 4–7; and 0.4%, VAS 8–10). At 30 days, mean (SD) VAS score was 0.5 (1.1), with 71% reporting a 0 score, 26.5% indicating a score of 1–3, 2.6% relating a 4–7 score, and no individuals reporting scores equal to or higher than 8. At 3 months, mean (SD) VAS remained low at 0.5 (1), with 78.5% reporting a 0 score, 18.4% indicating a score of 1–3, 3.1% relating a 4–7 score, and no individuals reporting scores equal to or higher than 8, suggesting early stabilization of pain at minimal levels. All data on postoperative VAS scores are outlined in (Table 3).

Post-hoc pairwise comparisons (reported in Figure 3) demonstrated significant reductions between discharge and 7–10 days, and further between 7–10 days and both 30 days and 3 months postoperatively, with no difference between the 30-day and 3-month assessments after adjustment for multiple testing. At 3 months, 8 cases (3.1%) reported a VAS score equal to or greater than 4, which serves as a pragmatic surrogate threshold approximating the protocol definition of clinically relevant persistent pain (VAS score >4 for over 3 months).

### Complications and additional interventions

The overall prevalence of postoperative complications was below 10% at each time point, occurring in 23 cases (8.1%) at 7–10 days, 24 (8.8%) at 30 days, and 20 (7.7%) at 3 months postoperatively, with no change over time (*P* = 0.64). At 7–10 days postsurgery, the most frequent complications were hematoma (56.5%) and seroma (34.8%), with few other events. At 30 days, seroma remained common (45.8%), while hematoma accounted for 12.5%, foreign-body sensation and testicular pain for 8%–12% each, and various other problems for 20.8%. At 3 months, the complication profile shifted toward symptom-based issues: intensified pain, groin discomfort, foreign-body sensation, and hydrocele each occurred in around 1% of all patients, with a small number of other events.

**TABLE 4:** Postoperative complications (n = 294)

Category		7–10 days postoperatively	30 days postoperatively	3 months postoperatively
Overall complications		23 (8.1)	24 (8.8)	20 (7.7)
Specific complications	Seroma	8 (34.8)	11 (45.8)	0
	Hematoma	13 (56.5)	3 (12.5)	2 (10)
	Testicular pain	0	2 (8.4)	0
	Foreign-body sensation	0	3 (12.5)	3 (15)
	Severe pain	0	0	3 (15)
	Groin discomfort	0	0	3 (15)
	Hydrocele	0	0	3 (15)
Additional interventions / unplanned visits		2 (0.7)	13 (4.8)	18 (6.9)

Data are presented are number (percentage).

Seroma incidence decreased between 30 days and 3 months postoperatively (*P* = 0.002). No significant change was observed for other specific complications over time. All data are presented in (Table 4).

### Hernia recurrence

No hernia recurrence was documented at any follow-up time point up to 3 months.

## DISCUSSION

The introduction of new surgical meshes into everyday practice has traditionally been driven largely by manufacturers, with surgeons often having to rely on information provided by distributors and sales representatives, occasionally supported by animal data but rarely by robust, independent clinical evidence in humans. The present study was therefore designed by the Hernia Chapter of the Association of Polish Surgeons as an independent, multicenter observational project to verify how a newly introduced implant, SWING-Mesh, behaves in everyday clinical use, and to provide surgeons and patients with transparent data on its effectiveness and safety rather than rely solely on industry-driven claims.

Our study shows that use of an unfixed, lightweight 3D SWING-Mesh implant in laparoendoscopic IH repair is associated with excellent early clinical outcomes. At 3 months, we observed no recurrences, very low pain scores, and a modest, mainly transient complication profile.

### Effectiveness and recurrence

The absence of any recurrence within 3 months postsurgery is a strong signal of early effectiveness. While true long-term hernia control requires extended observation, most technically failed repairs or gross mesh malposition manifest early. The upper 95% CI limit for recurrence in our cohort was around 1%, which lies well below the 2%–4% recurrence rates assumed in the sample size calculation based on historical laparoendoscopic series.

The decision to exclude large direct M3 hernias from this cohort also appears justified in hindsight. During the course of the study, an experimental pressure-chamber investigation evaluating the behavior of SWING-Mesh in an M3-type defect under increased intra-abdominal pressure demonstrated that the implant did not reliably maintain its intended position in this specific setting.^10^ These findings reinforce our protocol choice to avoid fixation-free use of this mesh in large direct hernias and support a more cautious, individualized approach for M3 defects. More broadly, recent work exploring technical modifications and mesh behavior in laparoendoscopic repair suggests that not every proposed adaptation translates into clinically meaningful benefit, underscoring the importance of independent real-world evidence and structured postmarket evaluation.^13^

Given that no additional fixation was used, these results support the concept that a properly sized anatomical 3D mesh can maintain stable positioning within the MPO solely by its shape and the surrounding tissue planes. This aligns with earlier clinical experience from other 3D meshes, particularly in large direct hernias, where fixation-free implantation did not increase recurrence. The planned 12-month follow-up will be essential to confirm if the favorable early recurrence profile is sustained over time, and to exclude late failures related to mesh shrinkage or progressive tissue changes.

### Pain and functional outcomes

Postoperative pain decreased rapidly and substantially in our cohort. Already at 7–10 days after surgery, over 95% of the patients had a VAS score equal to or below 3, and by 30 days, 71% were completely pain-free (VAS 0). At 3 months, nearly 80% reported no pain, and only 3.1% had a VAS score equal to or greater than 4.

These findings are clinically relevant in the context of chronic postherniorrhaphy pain, which is a key concern after groin surgery. The study protocol defined chronic pain as a VAS score above 4 persisting beyond 3 months and interfering with daily activities, with an acceptable threshold of 5% of the patients or fewer.^14^^,^^15^ Our early data fit comfortably within this target. Although we did not formally assess functional limitation at this stage, the low intensity of residual pain suggests that disabling chronic pain is unlikely to be common in this cohort.

Several factors may underlie these favorable pain outcomes: the minimally-invasive TAPP/TEP approaches, avoidance of rigid fixation devices, and the lightweight, macroporous design of SWING-Mesh, which is thought to reduce foreign-body reaction and stiffness. The observed low VAS scores and limited foreign-body sensation at 3 months postoperatively is consistent with this hypothesis, although definitive mechanistic conclusions cannot be drawn from an observational design.

Overall complication rates were below 10% at all time points, and most events were minor. Early postoperative complications were dominated by hematoma and seroma, which are common after IH repair, regardless of the technique used. By 3 months postsurgery, seromas had largely resolved, and the complication profile shifted to low-frequency symptom clusters, such as groin discomfort, foreign-body sensation, intensified pain, or hydrocele, each affecting around 1% of all patients. Importantly, only a small minority required additional postoperative interventions or unplanned visits (≤6.9% by 3 months after the procedure), suggesting a limited health care burden.

From a safety perspective, the data support that unfixed SWING-Mesh implantation is well tolerated in routine clinical practice. The complication rates observed compare favorably with published series of laparoendoscopic IH repair using standard meshes with or without fixation, although a direct comparison was not part of this study’s design.

### Strengths and limitations

The strengths of this study include its prospective design with a prespecified protocol and statistical analysis plan, a relatively large cohort (n = 294) enrolled from real-world specialized centers, and standardized data collection at multiple time points up to 3 months postoperatively. An additional strength is the focus on a single implant with clearly defined technical characteristics (3D, lightweight, microporous, and used without fixation), which reduces heterogeneity and allows for a more consistent interpretation of outcomes.

However, several limitations should also be acknowledged. First, the lack of a comparative control group means that the study is observational and single-arm. Effectiveness was assessed against historical benchmarks rather than a contemporaneous comparator. Second, the follow-up in this analysis is relatively short. Although 3 months is sufficient to characterize early pain and most early complications, it does not allow for a full evaluation of recurrence and chronic pain, and longer-term results—planned as a separate publication—are required. Third, there is potential center and surgeon variability: although all surgeons were experienced in laparoendoscopic repair, subtle differences in technique may have influenced outcomes, and detailed subgroup analyses (for example, TAPP vs TEP or BMI categories) were not presented in this early report. Finally, patient-reported outcome measures were limited, as pain was captured only via VAS, and no dedicated quality-of-life instruments or activity scales were used in this interim analysis. Although outcomes were analyzed at the operated-groin (case) level, a small subset of patients underwent bilateral repair and therefore contributed 2 cases, meaning that observations were not fully independent. However, this applied to only 11 out of 283 patients and is unlikely to have materially influenced the results. Nonetheless, it should be considered when interpreting case-level statistical comparisons.

Despite these limitations, the consistency of the findings—low pain intensity, low complication burden, and absence of recurrence at 3-month follow-up—strongly suggests that SWING-Mesh performs at least as well as established meshes in the short term, while potentially offering advantages related to its anatomical 3D design and fixation-free concept.

### Generalizability

The participation of a broad spectrum of hospitals in this study—including dedicated hernia centers, district hospitals, and specialist surgical units—supports the generalizability of our findings to a wider population of patients undergoing laparoendoscopic IH repair in routine clinical practice. This mix of high-volume referral centers and more general surgical departments reflects real-world conditions and increases the external validity of the results.

## CONCLUSIONS

In this prospective multicenter study of 294 cases undergoing laparoendoscopic IH repair, the use of an unfixed lightweight 3D SWING-Mesh implant was associated with no observed recurrence at 3-month follow-up (0%; 95% CI, up to approximately 1%), a rapid and sustained reduction in postoperative pain (with nearly 80% of the patients pain-free at 3 months postsurgery and only 3.1% reporting a VAS score ≥4), and low overall complication rates, with a very limited need for additional interventions. These early results indicate that SWING-Mesh is a safe and effective option for TAPP and TEP IH repair in routine clinical practice. Ongoing follow-up will clarify long-term recurrence and chronic pain outcomes, which will be reported in a separate 12-month analysis.

## JOURNAL INFORMATION

Videosurgery and Other Miniinvasive Techniques is an official journal of the Videosurgery Foundation.
